# Effectiveness of a Digital Health Game Intervention on Early Adolescent Smoking Refusal Self-Efficacy

**DOI:** 10.1177/10901981241237788

**Published:** 2024-03-18

**Authors:** Johanna Nyman, Sanna Salanterä, Miko Pasanen, Heidi Parisod

**Affiliations:** 1 University of Turku, Turku, Finland; 2 Turku University Hospital, Turku, Finland; 3 Nursing Research Foundation sr, Helsinki, Finland

**Keywords:** adolescents, adolescent health, health education, digital health, health games, health behavior

## Abstract

Smoking poses a significant threat to adolescent health because of its immediate and long-term detrimental health effects. Smoking refusal self-efficacy predicts smoking behavior in adolescence. In adolescents’ health education, digital interventions are potential tools to support smoking refusal self-efficacy. The aim of this two-arm cluster randomized controlled trial was to evaluate the effectiveness of a digital health game intervention compared with a no-intervention control group on smoking refusal self-efficacy in 10- to 13-year-old Finnish early adolescents. The early adolescents (*n* = 781) were randomized to the control group (*n* = 394) and the health game intervention group (*n* = 387). Smoking refusal self-efficacy, sources of smoking and snus refusal self-efficacy, and motivation to decline smoking and snus use in the future were measured at baseline, 2-week postintervention, and 3-month follow-up. Data were analyzed using linear mixed model and Wilcoxon rank-based test for clustered data. According to the results, the intervention group made improvements in sources of smoking and snus refusal self-efficacy between baseline and postintervention, and in sources of snus refusal self-efficacy between baseline and follow-up, compared with the control group. The intervention group showed improvements in smoking refusal self-efficacy among 12-year-olds between baseline and follow-up, and postintervention and follow-up compared with the control group. Similar improvements were also found among those with a smoking friend or a smoking parent between postintervention and follow-up. The results were promising for the use of digital health game interventions to promote early adolescent smoking refusal self-efficacy and preventing smoking experimentation. Further research can evaluate the long-term effects for adolescents.

## Introduction

Adolescents’ first smoking experimentations begin in early adolescence. For example, only 3.5% of 10- to 11-year-old Finnish adolescents had experimented with smoking in 2021, while the smoking experimentation rate among 14- to 15-year-olds was 35.9% (Finnish Institute for Health and Welfare, 2021a, 2021b). Among Finnish adolescents, also the use of snus has been common and increased in the 2010s, especially among boys (Kinnunen et al., 2019). Early smoking experimentation in particular leads to regular smoking (Sargent et al., 2017). Smoking poses a significant threat to the health of adolescents by exposing them to severe health risks during adolescence and later in life, such as respiratory infections and diseases, cancer, and cardiovascular disease (Dai et al., 2022).

Smoking refusal self-efficacy has been defined as the belief in one’s ability to refuse smoking (De Vries et al., 1988). Refusal self-efficacy predicts smoking behavior in adolescence (Lotrean et al., 2012; Wang et al., 2019), among other factors such as peer and parental norms and smoking behavior (O’Loughlin et al., 2017; Scalici & Schulz, 2017), perceived needs (O’Loughlin et al., 2017) as well as being offered cigarettes (Rachiotis et al., 2019). It has been suggested that effective smoking prevention interventions need to support adolescents’ social competence and refusal self-efficacy as well as their skills to resist social influences (Thomas et al., 2013).

Digital environments and gamification offer new possibilities for health promotion among adolescents, as playing digital games is popular among children and adolescents. In Finland, approximately 79% of adolescents aged 10 to 19 play digital games at least once a week, and the most popular gaming platform is mobile devices (77.4%), followed by computers (66.1%) and video game consoles (47.7%) (Kinnunen et al., 2020). Games, with their engaging and stimulating nature, have the potential to support behavior and attitude change by evoking strong emotions and requiring intricate cognitive processing (Boyle et al., 2011). In health education for children and adolescents, digital interventions present promising means to support their refusal self-efficacy (Nyman, Tornivuori, et al., 2022). The enactive and vicarious experiences provided by digital games can be successfully used to support adolescent self-efficacy by simulating real-life events with virtual game characters (Lee, 2015). Although more research on health games in adolescent health education and promotion is needed (Baranowski et al., 2016), the results of previous studies on game interventions to support nonsmoking have been encouraging in terms of adolescents’ interest (Parisod et al., 2018), attitudes (Parisod et al., 2018; Rath et al., 2015), and intentions (Andrews et al., 2014).

The aim of this study was to evaluate the effectiveness of a digital health game intervention compared with a no-intervention control group on smoking refusal self-efficacy in 10- to 13-year-old Finnish early adolescents. This age range was chosen to target the intervention toward early adolescents before their first tobacco product experimentations (Ma et al., 2021; Sharapova et al., 2020).

## Methods

### Study Design and Setting

**Figure 1.**
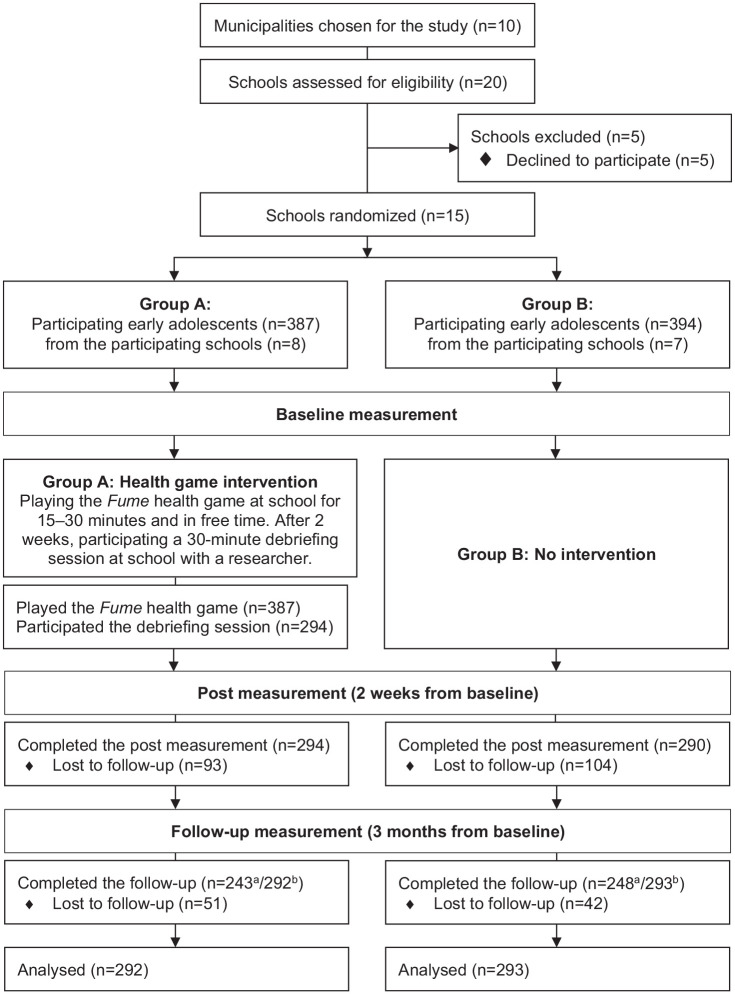
Study Design and Participant Flow. ^a^Completed measurement at all measurement points. ^b^Completed *measurement* at least at baseline and 3-month follow-up.

A two-arm cluster randomized controlled trial was conducted to be able to reach entire schools and the students in the schools (Brown et al., 2015). Outcomes were measured at 2-week postintervention and 3-month follow-up to be able to collect the data during the ongoing school year. Data were collected in 15 comprehensive schools from nine municipalities in Finland from February to May 2022. These municipalities represent areas in Finland where experimentation with tobacco products (cigarettes, snus, e-cigarettes) among early adolescents was the highest in 2021 (Finnish Institute for Health and Welfare, 2021a). All schools in these municipalities that met the following eligibility criteria were invited to participate in the study: (a) the school has at least one fourth, fifth, and sixth grade, (b) the official language is Finnish, and (c) the school has tablet computers for educational use. Participating schools were randomly allocated to two groups: the health game intervention group (Group A), and the no-intervention control group (Group B). Randomization was done at the school level to avoid contamination between the participants in the study groups. Computer-assisted randomization with random sequences was performed by a statistician not involved in school recruitment, with small village schools randomized to one stratum and all other schools to another stratum. The study design and participant flow are presented in more detail in Figure 1. The study was registered with the registration number NCT05290103 at ClinicalTrials.gov.

**Impact Statement**

This study supports the implementation of digital health game interventions for adolescents’ health education to support smoking refusal self-efficacy, and thus, prevent smoking experimentation. The Fume health game intervention was effective in supporting early adolescent smoking refusal self-efficacy among 12-year-olds, and those who had people who smoke in their immediate social circle. The Fume health game also supported early adolescents’ sources of smoking and snus refusal self-efficacy among all participants. However, the appropriate duration of interventions supporting smoking refusal self-efficacy among adolescents and their content on newer tobacco and nicotine products need to be considered.

### Participants

The sample size was determined in advance with a power analysis based on our previous study (Parisod et al., 2018). With an effect size of 0.30, alpha of 0.05, and power of 0.80, the estimated total sample size required was 290. However, cluster randomized trials require more participants (Puffer et al., 2005), and thus, the clustering effect of 1.49 (based on the estimated ICC = 0.01, and cluster size = 50) was used to determine the sample size. With an estimated loss of 20%, the total sample size required was 542 early adolescents (271 in each group).

All students in the participating schools were invited to participate in the study if they met the eligibility criteria: (a) were in grades fourth, fifth, and sixth, and (b) had sufficient ability to understand and speak Finnish, Swedish, or English, and (c) were willing to participate in the study. Participants were excluded from the study if their parents or guardians refused their participation. At the 2-week postmeasurement, there was a 25% loss of participants due to the COVID-19 epidemic and absences from school, and problems with e-mail. Some students had accidentally or intentionally given a false e-mail address or no e-mail address at all, or were unable to log on to their e-mail. At the 3-month follow-up, there was a 16% loss from the 2-week postmeasurement due to absences from school and problems with logging on to e-mail. However, no entire schools dropped out of the study at either the postmeasurement or the follow-up measurement.

### Intervention

The health game intervention group received an intervention consisting of a digital health game and a debriefing session after game-play. The control group received no intervention.

The digital health game is called Fume (see Supplementary file 1), and it was developed to support health literacy and self-efficacy related to early adolescent nonsmoking (Parisod et al., 2017). Fume is a mobile game that includes seven different mini-games in which players learn about the consequences of smoking and snus use on their health, physical condition, personal finances, and the environment. They can also practice refusing to smoke and use snus. The early adolescents could play Fume either via tablet computers, smartphones, or computers (using web browsers). The preliminary effectiveness of the Fume game has been studied in a pilot study with promising results on attitudes toward smoking and smoking outcome expectations (Parisod et al., 2018). In this study, Fume was further developed to better support early adolescents’ refusal self-efficacy related to smoking and snus use based on the self-efficacy theory (Bandura, 1997), for example, by placing more emphasis on game elements in which early adolescents get to practice resisting peer influence and refusing cigarettes and snus.

Based on the pilot study, the Fume game was complemented with a debriefing session to further support early adolescents’ learning by linking the game experiences to their real-life situations and events (Garris et al., 2002; Hromek & Roffey, 2009). In the debriefing sessions, the early adolescents were asked to discuss their game experiences in small groups and whole class groups, and to reflect on the positive sides of tobacco- and snus-free life and how to refuse to smoke and use snus. The debriefing sessions were held by a researcher via video conferencing platforms to each class in each school in the intervention group. Teachers facilitated the sessions by opening the remote connection, assisting students in dividing into small groups, and ensuring a good learning environment.

After baseline, the participants played Fume at school for 15 to 30 minutes. During the study period, they could also play the game in their free time according to their own choice. After 2 weeks, a 30-minute debriefing session was held for each class.

### Outcome Measures

#### Background Information

The early adolescents completed a background information questionnaire developed for this study at baseline. The electronic questionnaire consisted of items on participant demographics (such as age and gender) and items related to adolescent smoking refusal self-efficacy based on previous studies (such as experiences with cigarettes and snus).

#### Effectiveness

The early adolescents completed instruments via electronic questionnaires on smoking refusal self-efficacy and secondary outcomes at baseline, 2-week postintervention, and 3-month follow-up. Smoking refusal self-efficacy was considered as the primary outcome and was measured using a previously developed and tested instrument (Lazuras et al., 2009). Secondary outcomes were sources of smoking refusal self-efficacy, sources of snus refusal self-efficacy, motivation to decline smoking in the future, and motivation to decline snus use in the future (see Table 1).

**Table 1.** Outcome Measures and Instruments.

Instrument	Response format	Items	Possible range	α^a^
Smoking refusal self-efficacy (Lazuras et al., 2009)	4-point Likert scale (1 = strongly disagree, 4 = strongly agree)	Six items on adolescent self-efficacy in refusing smoking in different situations; for example, “I can say no to smoking, when my friends want me to smoke”	6–24 (sum), higher scores indicate higher levels of smoking refusal self-efficacy	T0: 0.89 T1: 0.92 T2: 0.91
Sources of smoking refusal self-efficacy^b^	4-point Likert scale (1 = bad, 4 = good; 1 = easy, 4 = difficult; 1 = disagree, 4 = agree)	Seven items out of which one item measures mastery experiences, four items vicarious experiences, one item social persuasion, and one item physiological and emotional states related to smoking refusal self-efficacy; for example, “I feel that people around me find smoking acceptable”,	7–28 (sum, five items reverse-coded), higher scores indicate stronger sources of self-efficacy related to smoking refusal	T0: 0.61 T1: 0.66 T2: 0.71
Sources of snus refusal self-efficacy^b^	4-point Likert scale (1=bad, 4=good; 1=easy, 4=difficult; 1=disagree, 4=agree)	Seven items out of which one item measures mastery experiences, four items vicarious experiences, one item social persuasion, and one item physiological and emotional states related to snus refusal self-efficacy, for example, “I feel that people around me find snus use acceptable”.	7–28 (sum, five items reverse-coded), higher scores indicate stronger sources of self-efficacy related to snus refusal	T0: 0.65 T1: 0.67 T2: 0.71
Motivation to decline cigarette smoking in the future (Parisod et al., 2018)	4-point Likert scale (1 = I’m sure I couldn’t, 4 = I’m sure I could)	One item on motivation to decline cigarette smoking in the future	1–4 (item reverse-coded), a higher score indicates higher motivation to decline cigarette smoking in the future	NA
Motivation to decline snus use in the future (Parisod et al., 2018)	4-point Likert scale (1 = I’m sure I couldn’t, 4 = I’m sure I could)	One item on motivation to decline snus use in the future	1–4 (item reverse-coded), a higher score indicates higher motivation to decline snus use in the future	NA

NA = not available, Cronbach’s alpha could not be calculated with one-item instrument.

T0 = baseline measurement, T1 = 2-week post measurement, T2 = 3-month follow-up measurement.

a Cronbach’s alpha indicates internal consistency of the instrument.

b Instrument pilot tested and developed for this study based on previous studies and the Self-Efficacy Theory (Bandura, 1997).

### Data Analysis

Data were analyzed using R version 4.0.2. Descriptive statistics (mean, percentage) were used to describe the sample. Differences between the two groups were examined using Fisher’s Exact test and Mann Whitney U-test. The same tests were used to test differences between those dropping out of the study at postintervention and those continuing to participate. Inferential statistics were used to evaluate the effectiveness of the digital health game intervention on early adolescents’ smoking refusal self-efficacy, sources of smoking and snus refusal self-efficacy, and motivation to decline cigarette smoking and snus use in the future. Effectiveness was evaluated by testing for changes between the two study groups and within the groups. A linear mixed model (LMM) was used to analyze the intervention effects both between and within the two study groups within the three time points. Age and gender were selected as fixed controlled factors, and schools were selected as random effects to account for cluster effects. Subgroup analyses based on age, gender, friends’ and parents’ smoking behavior, smoking experiments, and lower scores were conducted using interaction terms (study group, time point, and subgroup). For individual items, such as motivation to decline cigarette smoking and snus use in the future, the Wilcoxon rank-based test for clustered data (R package clusrank version 1.0-3) was used to test for change between time points, with school as a clustering variable (Jiang et al., 2020). The reliability of the scales was evaluated using Cronbach’s alpha.

### Ethical Considerations

Ethical approval was obtained from the Ethics Committee for Human Sciences at the University of Turku (reference numbers 27/2021 and 2/2022). The students and their parents or guardians were approached before data collection and given information about the study. As the participants were children under the age of 15, the parents or guardians could refuse their child’s participation (Finnish National Board on Research Integrity TENK, 2019). The early adolescents gave their consent to participate in the study by completing the electronic questionnaires and could terminate their participation at any time. Permission to use the instruments used to measure outcomes was obtained from the authors.

## Results

### Participant Characteristics

A total of 781 early adolescents participated in the study at baseline, 584 at the 2-week postmeasurement, and 585 at the 3-month follow-up (of whom 491 participated at all measurement points) (see Figure 1). The mean age of the participating early adolescents was 11.25 years. Baseline background characteristics of the early adolescents are presented in Table 2. The two groups were rather similar based on their background characteristics, but there was one statistically significant difference, that is, grandparents’ cigarette smoking (*p* = .032).

**Table 2.** Background Characteristics of the Early Adolescents.

Background variable	All participants (*n* = 781)	Health game intervention group (*n* = 387)	Control group (*n* = 394)	*p* value^a^
Age, mean	11.25	11.27	11.24	0.949
10 or younger, *n* (%)	187 (24.2)	90 (23.3)	97 (25.1)	
11, *n* (%)	252 (32.6)	127 (32.9)	125 (32.3)	
12, *n* (%)	282 (36.5)	142 (36.8)	140 (36.2)	
13 or older, *n* (%)	52 (6.7)	27 (7.0)	25 (6.5)	
Gender				0.481
Female, *n* (%)	367 (47.4)	182 (47.0)	185 (47.7)	
Male, *n* (%)	362 (46.7)	182 (47.0)	180 (46.4)	
Other, *n* (%)	15 (1.9)	10 (2.6)	5 (1.3)	
Doesn’t want to answer, *n* (%)	31 (4.0)	13 (3.4)	18 (4.6)	
Native language: Finnish, *n* (%)	755 (98.3)	377 (98.2)	378 (98.4)	0.611
Mother’s native language: Finnish, *n* (%)	744 (97.0)	372 (97.6)	372 (96.4)	0.279
Father’s native language: Finnish, *n* (%)	742 (96.7)	373 (97.4)	369 (96.1)	0.146
Has been offered cigarettes, *n* (%)	73 (9.4)	31 (8.0)	42 (10.8)	0.217
Has tried smoking cigarettes, *n* (%)	46 (5.9)	21 (5.4)	25 (6.4)	0.649
Smokes cigarettes currently, *n* (%)	9 (1.2)	2 (0.5)	7 (1.8)	
Has been offered snus, *n* (%)	50 (6.5)	24 (6.2)	26 (6.7)	0.884
Has tried using snus, *n* (%)	28 (3.6)	16 (4.1)	12 (3.1)	0.564
Uses snus currently, *n* (%)	5 (0.6)	1 (0.3)	4 (1.0)	
Cigarette smoking of others				
Parents, *n* (%)	274 (35.1)	140 (36.2)	134 (34.0)	0.549
Siblings, *n* (%)	91 (11.7)	42 (10.9)	49 (12.4)	0.505
Grandparents, *n* (%)	126 (16.1)	51 (13.2)	75 (19.0)	0.032
Friends, *n* (%)	68 (8.7)	24 (6.2)	33 (8.4)	0.272
Authorities (e.g. teacher and sports coach), *n* (%)	80 (10.2)	32 (8.3)	33 (8.4)	1
Idols, *n* (%)	46 (5.9)	22 (5.7)	24 (6.1)	0.880
Snus use of others				
Parents, *n* (%)	72 (9.2)	39 (10.1)	33 (8.4)	0.459
Siblings, *n* (%)	58 (7.4)	35 (9.0)	23 (5.8)	0.102
Grandparents, *n* (%)	11 (1.4)	5 (1.3)	6 (1.5)	1
Friends, *n* (%)	47 (6.0)	20 (5.2)	23 (5.8)	0.755
Authorities (e.g. teacher and sports coach), *n* (%)	28 (3.6)	4 (1.0)	8 (2.0)	0.384
Idols, *n* (%)	23 (2.9)	15 (3.9)	8 (2.0)	0.142
Frequently sees other people smoking cigarettes, *n* (%)	448 (57.9)	228 (58.9)	220 (56.8)	0.610
Frequently sees other people using snus, *n* (%)	132 (17.7)	69 (18.4)	63 (17.0)	0.632
Reports having received health education about cigarettes during current school year, *n* (%)	358 (46.7)	179 (46.9)	179 (46.6)	1
Reports having received health education about snus during current school year, *n* (%)	276 (36.1)	147 (38.4)	129 (33.8)	0.201
Values				
Athletic achievements, *n* (%)	312 (39.9)	157 (40.6)	155 (39.3)	0.770
Nature conservation, *n* (%)	329 (42.1)	158 (40.8)	171 (43.4)	0.470
Money, *n* (%)	243 (31.1)	121 (31.3)	122 (31.0)	0.939
Health, *n* (%)	650 (83.2)	328 (84.8)	322 (81.7)	0.292
Friends’ acceptance, *n* (%)	457 (58.5)	231 (59.7)	226 (57.4)	0.514
Looking good, *n* (%)	90 (11.5)	51 (13.2)	39 (9.9)	0.179
Looking cool, *n* (%)	34 (4.4)	18 (4.7)	16 (4.1)	0.728
Looking grown-up, *n* (%)	13 (1.7)	4 (1.0)	9 (2.3)	0.263
Looking tough, *n* (%)	23 (2.9)	10 (2.6)	13 (3.3)	0.673

a P value describes differences between the health game intervention group and the control group.

### Effectiveness of the Intervention

The results of the LMM analysis of early adolescents’ smoking refusal self-efficacy and sources of refusal self-efficacy related to smoking and snus refusal are presented in Table 3.

**Table 3.** Results of the Linear Mixed Model Analysis Related to Smoking Refusal Self-Efficacy as Well as Sources of Smoking and Snus Refusal Self-Efficacy.

	Within group						Between group (group X time)
	Control			Intervention			
	Mean (*SD*)^a^ change in mean^b^	LMM estimate (95% CI)	P value	Mean (*SD*)^a^ change in mean^b^	LMM estimate (95% CI)	P value	P value
Smoking refusal self-efficacy							
Baseline	23.05 (2.13)			22.87 (3.01)			
Time (T1–T0)	−0.15	−0.151 (−0.488, 0.187)	0.548	−0.29	−0.298 (−0.636, 0.040)	0.097	0.851
Time (T2–T0)	−0.48	−0.556 (−0.913, −0.199)	0.001**	−0.09	−0.249 (−0.612, 0.114)	0.241	0.402
Time (T2–T1)	−0.33	−0.405 (−0.762, −0.0483)	0.021*	0.20	0.049 (−0.314, 0.411)	0.946	0.105
Sources of smoking refusal self-efficacy							
Baseline	23.02 (3.15)			23.15 (3.06)			
Time (T1–T0)	−0.29	−0.282 (−0.647, 0.083)	0.166	0.22	0.247 (−0.121, 0.615)	0.258	0.0499*
Time (T2–T0)	−0.45	−0.609 (−0.995, −0.223)	0.001**	−0.10	−0.217 (−0.610, 0.175)	0.395	0.259
Time (T2–T1)	−0.16	−0.327 (−0.713, 0.059)	0.115	−0.32	−0.464 (−0.855, −0.073)	0.015*	0.914
Sources of snus refusal self-efficacy							
Baseline	23.01 (3.39)			23.02 (3.19)			
Time (T1–T0)	−0.21	−0.251 (−0.618, 0.117)	0.245	0.40	0.400 (0.031, 0.770)	0.030*	0.010*
Time (T2–T0)	−0.31	−0.527 (−0.915, −0.139)	0.004**	0.16	0.079 (−0.314, 0.472)	0.885	0.030*
Time (T2–T1)	−0.10	−0.276 (−0.664, 0.111)	0.216	−0.24	−0.321 (−0.715, 0.072)	0.134	0.996

T0 = baseline measurement, T1 = 2-week post measurement, T2 = 3-month follow-up measurement.

SD = standard deviation, LMM = linear mixed model, CI = confidence interval.

a Mean value and standard deviation for baseline measurement.

b Change in mean between the time points.

* *p* < .05, **p < .01.

#### Smoking Refusal Self-Efficacy

In the control group, the smoking refusal self-efficacy scores decreased, indicating weaker self-efficacy at 2-week postintervention and 3-month follow-up. The change was statistically significant between baseline and 3-month follow-up (*p* = .001) and between 2-week postintervention and 3-month follow-up (*p* = .021). Also, in the intervention group, the smoking refusal self-efficacy scores decreased at 2-week postintervention, but increased slightly at 3-month follow-up. However, these changes were not statistically significant. In addition, there were no statistically significant differences between the two groups in the changes of smoking refusal self-efficacy scores at any time point.

#### Sources of Smoking Refusal Self-Efficacy

In the control group, the sources of smoking refusal self-efficacy scores decreased at all time points. The decrease was statistically significant between baseline and 3-month follow-up (*p* = .001). In the intervention group, the sources of smoking refusal self-efficacy scores improved slightly between baseline and 2-week postintervention, but the change was not statistically significant. At the other time points, scores decreased and were statistically significant between 2-week postintervention and 3-month follow-up (*p* = .015). However, compared with the control group, the intervention group made statistically significant improvements in sources of smoking refusal self-efficacy among the participating early adolescents between baseline and 2-week postintervention (*p* = .0499).

#### Sources of Snus Refusal Self-Efficacy

Sources of snus refusal self-efficacy scores of the early adolescents in the control group decreased at all time points, with statistical significance between baseline and 3-month follow-up (*p* = .004). However, in the intervention group, the scores of the sources of snus refusal self-efficacy improved with statistical significance between baseline and 2-week postintervention (*p* = .030) and improved slightly, but not with statistical significance, between baseline and 3-month follow-up. When examining between-group results, the intervention group showed statistically significant improvements between baseline and 2-week postintervention (*p* = .010) and baseline and 3-month follow-up (*p* = .030) relative to the control group.

#### Motivation to Decline Cigarette Smoking and Snus Use in the Future

There were no statistically significant within-group or between-group changes in early adolescents’ motivation to decline cigarette smoking and snus use in the future at any time point (see Table 4).

**Table 4.** Outcome Values for the Motivation to Decline Cigarette Smoking and Snus Use in the Future at Baseline, After 2 Weeks, and After 3 Months.

	Within group				Between group
	Control group		Intervention group		
	Mean (*SD*)	*p* value	Mean (*SD*)	*p* value	*p* value
Motivation to decline cigarette smoking in the future					
T0	3.55 (0.71)		3.63 (0.64)		
T1	3.53 (0.77)	0.685	3.70 (0.57)	0.799	0.645
T2	3.53 (0.75)	0.433	3.68 (0.61)	0.376	0.242
Motivation to decline snus use in the future					
T0	3.75 (0.61)		3.78 (0.56)		
T1	3.76 (0.56)	0.805	3.80 (0.55)	0.791	1.000
T2	3.76 (0.56)	0.372	3.81 (0.52)	0.685	0.354

T0 = baseline measurement, T1 = 2-week postmeasurement, T2 = 3-month follow-up measurement.

SD = standard deviation.

#### Supplementary Analyses

The results of the subgroup analyses showed that (see Supplementary file 2), compared with the control group, the intervention group made statistically significant improvements among of 12-year-olds in smoking refusal self-efficacy between baseline and 3-month follow-up (*p* = .028) and 2-week postintervention and 3-month follow-up (*p* = .041) and in sources of snus refusal self-efficacy between baseline and 3-month follow-up (*p* = .004). Among 9- to 10-year-olds, the intervention group showed statistically significant improvements in sources of smoking refusal self-efficacy between baseline and 2-week postintervention relative to the control group (*p* = .019).

In the subgroup analysis based on gender, the early adolescents in the intervention group who reported their gender as “other” or did not want to report their gender showed improvements in smoking refusal self-efficacy between 2-week postintervention and 3-month follow-up compared with the control group (*p* = .003). The same early adolescents showed statistically significant decrease in the smoking refusal self-efficacy scores between baseline and 2-week postintervention relative to the control group (*p* = .004). However, the results need to be treated with caution due to a small sample size. Regarding the sources of smoking and snus refusal self-efficacy, the intervention group made statistically significant improvements between baseline and 2-week postintervention compared with the control group among female participants (smoking: *p* = .007, snus: *p* = .006).

Relative to the control group, the early adolescents in the intervention group who had a smoking friend or a smoking parent showed statistically significant improvements in smoking refusal self-efficacy between 2-week postintervention and 3-month follow-up (smoking friend: *p* = .028, smoking parent: *p* = .033). However, the results concerning smoking friends need to be treated with caution due to a small sample size. The intervention group made statistically significant improvements between baseline and 2-week postintervention on sources of smoking refusal self-efficacy compared with the control group among participants who reported that their friends or parents did not smoke (nonsmoking friends: *p* = .027, nonsmoking parents: *p* = .0098). Similar improvements were found in sources of snus refusal self-efficacy at the same time point (nonsmoking friends: *p* = .016, nonsmoking parents: *p* < .001).

Among the early adolescents who had tried smoking, there were no statistically significant differences in the changes in smoking refusal self-efficacy scores between the two groups. However, compared with the control group, the early adolescents in the intervention group who had not tried smoking showed statistically significant improvements between baseline and 2-week postintervention in sources of smoking (*p* = .031) and snus (*p* = .019) refusal self-efficacy.

Among early adolescents with lower baseline scores (the lowest 10% due to a skewed distribution), there were no statistically differences between the two groups in smoking refusal self-efficacy and sources of smoking refusal self-efficacy at any time point. However, relative to the control group, the intervention group made statistically significant improvements in sources of snus refusal self-efficacy among participants with lower scores (the lowest 25%) between baseline and 2-week postintervention (*p* = .011) and baseline and 3-month follow-up (*p* = .031).

## Discussion

This study examined the effectiveness of a digital health game intervention on smoking refusal self-efficacy and related factors in early adolescents. According to the results, the intervention has positive effects at 3 months on smoking refusal self-efficacy especially among 12-year-olds and those who had a smoking friend or a smoking parent. In addition, the intervention had favorable effects on the sources of smoking and snus refusal self-efficacy at postintervention.

In this study, the results on smoking refusal self-efficacy were most positive among 12-year-old early adolescents. The smoking refusal self-efficacy levels of the early adolescents in the control group decreased most among 12-year-olds. This was also the only age group in which the health game intervention had statistically significant favorable effects on smoking refusal self-efficacy. Thus, the intervention seems to be most suitable for 12-year-old early adolescents. However, the intervention and its content were designed to be age-appropriate for early adolescents, and tailoring interventions for each age specifically is difficult and likely not cost-effective. This age, when early adolescents transition into adolescence and tobacco-related issues become more topical, may also be a critical age in the development of smoking refusal self-efficacy and from the perspective of experimenting with tobacco products. For example, previous research indicates that a significant proportion of adolescents experiment with tobacco and nicotine products in early adolescence (Macron et al., 2018), specifically by the age of 13 years (Sharapova et al., 2020).

Previous studies have shown that social pressure, especially from peers, is crucial in terms of adolescents’ refusal self-efficacy and health behaviors related to tobacco product or substance use (Gázquez Linares et al., 2023; Moilanen et al., 2021; Nyman, Pinto, et al., 2022). Also, in this study, the intervention had favorable effects on smoking refusal self-efficacy among early adolescents who had a smoking friend or a smoking parent. This suggests that the intervention may be most effective among those most at risk of social pressure or influence. Interestingly, the results of the intervention on sources of smoking and snus refusal self-efficacy were most positive among early adolescents whose parents or friends did not smoke. This may indicate that the intervention was also effective in strengthening the positive feelings related to not smoking and not using snus among those who faced less social pressure or influence to use tobacco products.

Although not all results were statistically significant, there was a trend in the scores of the early adolescents’ smoking refusal self-efficacy and related sources during the study. Among early adolescents in the control group, all scores decreased from baseline at postintervention and 3-month follow-up. However, among participants who received the health game intervention, the smoking refusal self-efficacy scores decreased at postintervention and then increased slightly at follow-up. However, the scores related to sources of smoking and snus refusal self-efficacy increased in the intervention group at postintervention. According to the Self-Efficacy Theory, self-efficacy beliefs are developed through the four sources of self-efficacy (mastery experiences, vicarious experiences, social persuasion, and physiological and emotional states) (Bandura, 1997). Thus, the results are consistent with previous literature, as it is assumed that changes in early adolescents’ sources of refusal self-efficacy are more susceptible to change and influence the level of refusal self-efficacy later on. However, the results showed that the intervention did not steadily improve the sources of smoking and snus refusal self-efficacy, but decreased at follow-up. This indicates that there may be a need for a recurrent intervention or booster sessions on the intervention that are sustained over a longer period of time.

The baseline smoking refusal self-efficacy scores of the early adolescents were high indicating that they already had a strong self-efficacy to refuse smoking from the beginning. It seems that many Finnish early adolescents believe in their ability to refuse smoking, and that they can and have the courage to stick to their opinions in situations of social pressure or influence. Another study from Finland also found that health choices are a way for adolescents to demonstrate their individuality (Moilanen et al., 2021). In other cultures, adolescents’ refusal self-efficacy may be more strongly related to, for example, behavioral control by parents (Wang et al., 2019).

Because of the high baseline scores, there was not much room for the scores to improve at later measurement points, making it difficult to detect positive changes in smoking refusal self-efficacy among early adolescents. Nevertheless, there were improvements in smoking refusal self-efficacy among the early adolescents in the intervention group at follow-up and statistically significant improvements in some subgroup analyses. In light of previous literature, even these small improvements in smoking refusal self-efficacy can be considered significant in terms of adolescent smoking prevention. For example, according to a longitudinal study by Hiemstra et al. (2011) decreases in smoking refusal self-efficacy over time are particularly concerning, because they are associated with smoking initiation. Thus, implementation of the Fume health game intervention, which requires little effort, in health education may be beneficial to support early adolescent smoking refusal self-efficacy by preventing decreases in self-efficacy over time.

In this study, improvements in the study outcomes were greatest in terms of sources of snus refusal self-efficacy. Recent studies have shown that adolescents understand the harms of tobacco better than, for example, snus or newer tobacco and nicotine products, such as e-cigarettes or waterpipes (El-Amin et al., 2022; Scheffels et al., 2023). Thus, the Fume health game intervention may be more effective with respect to newer tobacco and nicotine products, the harmful health effects of which are less well understood by adolescents. At the same time, adolescent experimentation with snus and e-cigarettes is common in the Nordic countries (Cerrai et al., 2022; El-Amin et al., 2022; Raitasalo et al., 2022). This suggests that there may be a need to further strengthen the health education perspective on newer tobacco and nicotine products in the Fume intervention.

Self-reported instruments were used to measure intervention outcomes, and baseline levels of smoking refusal self-efficacy were high. Thus, the early adolescents may have overestimated their smoking refusal self-efficacy. However, this risk is similar in both arms and another instrument measuring sources of self-efficacy related to smoking refusal was used to control for this risk. Two instruments were developed and pre-tested for this study, and their internal consistency was slightly low at baseline indicating a need for further development. However, at the 3-month follow-up, the internal consistencies were satisfactory for comparing groups (Bland & Altman, 1997). In addition to these two instruments, a previously developed and used instrument with good internal consistency was used to measure the primary outcome. The Fume game was freely available on application stores, and thus there was a risk of contamination. To identify and control for this risk, the early adolescents were asked at the 3-month follow-up if they had played the Fume game before participating in the study. Only two early adolescents in the control group reported having played the Fume game. Upload and usage rates of the game were also tracked during the study period by following the game analytics in Finland. Since playing the game did not require registration, we could not track individual early adolescents gaming frequency. However, the upload and usage rates remained similar to those prior to the study suggesting that the intervention did not significantly increase the use of the mobile game application outside of the intervention. Most of the intervention schools played the Fume game through a web link distributed only to the intervention schools. There is also a risk of confounding factors (such as out-of-school health education) that may have influenced the results in addition to the intervention. However, this risk is similar in both arms, and the schools in both arms were similar as they were all public schools following the national core curricula. There was a 37% loss of early adolescents from baseline to 3-month follow-up. However, the baseline sample size was large, and the loss was considered in advance in the power analysis. Also, the reasons and numbers of early adolescents dropping out of the study were similar in both arms. The results can be generalized to similar contexts and to adolescents with similar backgrounds.

## Conclusion

The Fume health game intervention is an effective method to support early adolescent smoking refusal self-efficacy. Results were positive on the sources of smoking and snus refusal self-efficacy, and on smoking refusal self-efficacy especially among 12-year-olds, and those who had people who smoke in their immediate social circle. This study supports the implementation of digital health game interventions for health education in children and adolescents, for example in school health care, to support smoking refusal self-efficacy, and thus, prevent smoking experimentation. Further research is needed to evaluate the long-term effects of the intervention, to consider the appropriate duration of the intervention, and content on newer tobacco and nicotine products.

## Supplemental Material

sj-docx-1-heb-10.1177_10901981241237788 – Supplemental material for Effectiveness of a Digital Health Game Intervention on Early Adolescent Smoking Refusal Self-Efficacy Supplemental material, sj-docx-1-heb-10.1177_10901981241237788 for Effectiveness of a Digital Health Game Intervention on Early Adolescent Smoking Refusal Self-Efficacy by Johanna Nyman, Sanna Salanterä, Miko Pasanen and Heidi Parisod in Health Education & Behavior

sj-docx-2-heb-10.1177_10901981241237788 – Supplemental material for Effectiveness of a Digital Health Game Intervention on Early Adolescent Smoking Refusal Self-Efficacy Supplemental material, sj-docx-2-heb-10.1177_10901981241237788 for Effectiveness of a Digital Health Game Intervention on Early Adolescent Smoking Refusal Self-Efficacy by Johanna Nyman, Sanna Salanterä, Miko Pasanen and Heidi Parisod in Health Education & Behavior
