# Supplementary material for: Effectiveness of a Digital Health Game Intervention on Early Adolescent Smoking Refusal Self-Efficacy
Source: Health Educ Behav. 2024 Mar 18;51(4):562–72. doi: 10.1177/10901981241237788 (PMC11193316; doi:10.1177/10901981241237788)
Supplement: sj-docx-1-heb-10.1177_10901981241237788 – Supplemental material for Effectiveness of a Digital Health Game Intervention on Early Adolescent Smoking Refusal Self-Efficacy [file sj-docx-1-heb-10.1177_10901981241237788.docx]

**Supplementary file 1**

Description of the goals and rules of the *Fume* game

The *Fume* game consists of seven mini-games each featuring a task for the player. Each mini-game may appear multiple times in a random order during one gaming session. The goal of the game is that the players accomplish tasks in the minigames within a set time limit. At the first level, the time limit varies from around 10 to 30 seconds, but as the player proceeds, the time limit shortens. The player is given three lives for each gaming session. Scores in the game are assigned based on the player's performance and the choices they make. (Parisod et al., 2017)

Screenshot images of the *Fume* game (Image 1, 2, and 3).


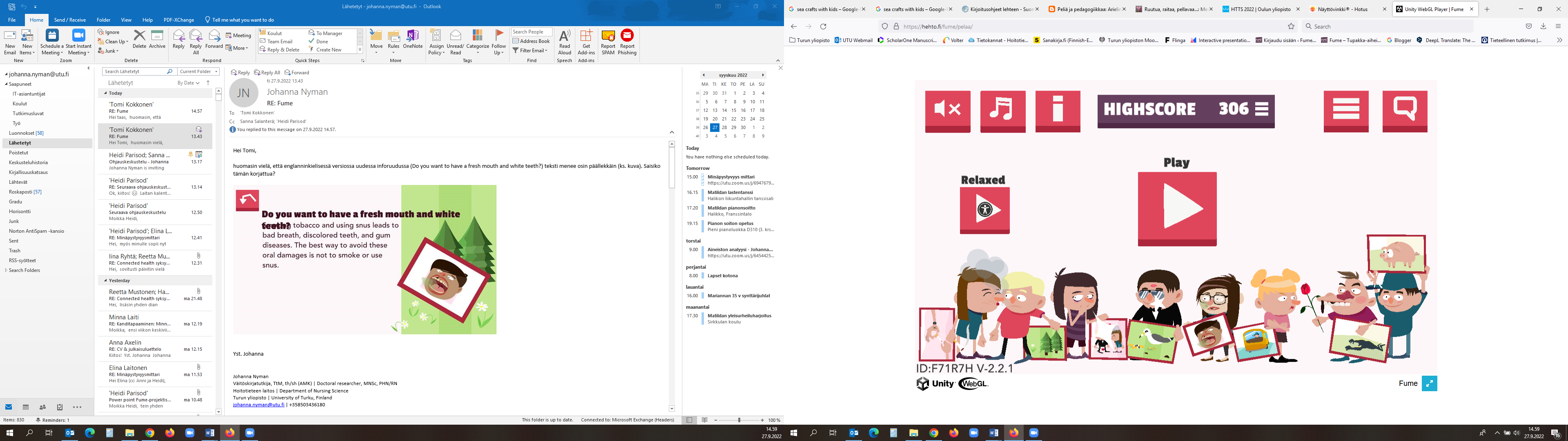
Image 1. Start screen of the *Fume* game (I am the copyright owner of this picture).

Image 2. ‘Refusing tobacco and snus’ mini-game (I am the copyright owner of this picture).


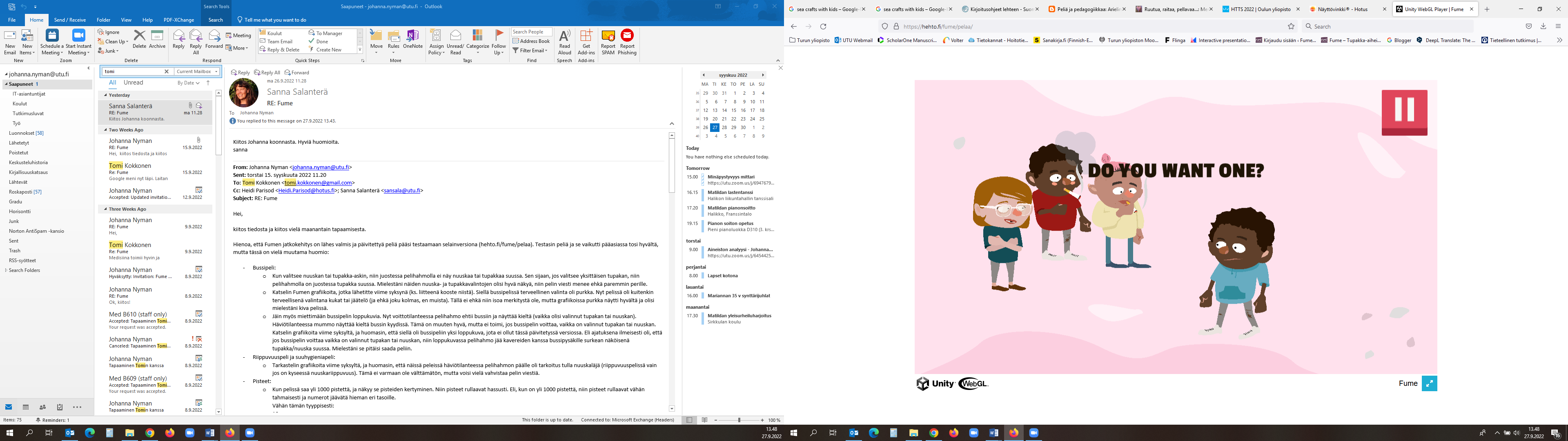


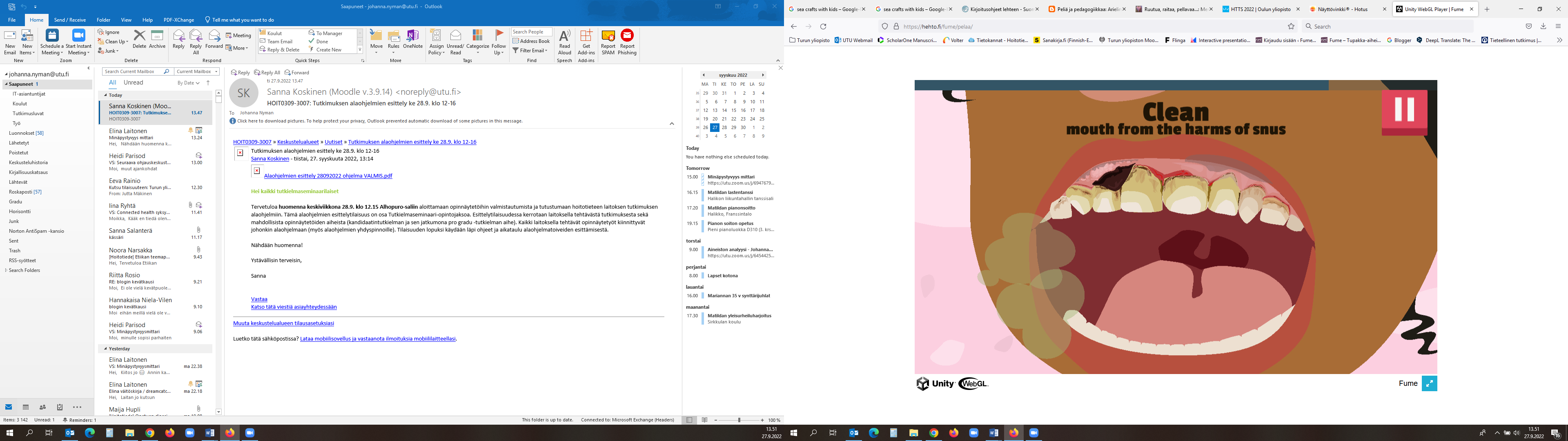
Image 4. ‘Harms of snus use’ mini-game (I am the copyright owner of this picture).
