# Supplementary material for: Effectiveness of a Digital Health Game Intervention on Early Adolescent Smoking Refusal Self-Efficacy
Source: Health Educ Behav. 2024 Mar 18;51(4):562–72. doi: 10.1177/10901981241237788 (PMC11193316; doi:10.1177/10901981241237788)
Supplement: sj-docx-2-heb-10.1177_10901981241237788 – Supplemental material for Effectiveness of a Digital Health Game Intervention on Early Adolescent Smoking Refusal Self-Efficacy [file sj-docx-2-heb-10.1177_10901981241237788.docx]

**Supplementary file 2**

Table 1. Subgroup analysis on smoking refusal self-efficacy among early adolescents

|  | Within group | | | | | | |  | | Between group (Group X Time) | |  |
| --- | --- | --- | --- | --- | --- | --- | --- | --- | --- | --- | --- | --- |
|  | Control | | |  | Intervention | | |  | |  | |  |
|  | Mean (SD) | LMM estimate (95% CI) | P value |  | Mean (SD) | LMM estimate (95% CI) | P value | |  | | P value | |
|  |  |  |  |  |  |  |  | |  | |  | |
| Age: 9-10 years |  |  |  |  |  |  |  | |  | |  | |
| Baseline | 23.37 (1.72) |  |  |  | 23.21 (3.01) |  |  | |  | |  | |
| Time (T1-T0) |  | -0.139 (-0.780, 0.502) | 0.867 |  |  | -0.754 (-1.439, -0.069) | **0.027*** | |  | | 0.328 | |
| Time (T2-T0) |  | -0.359 (-1.024, 0.307) | 0.415 |  |  | -0.471 (-1.183, 0.242) | 0.268 | |  | | 0.990 | |
| Time (T2-T1) |  | -0.220 (-0.885, 0.446) | 0.719 |  |  | 0.283 (-0.432, 0.999) | 0.621 | |  | | 0.538 | |
| Age: 11 years |  |  |  |  |  |  |  | |  | |  | |
| Baseline | 23.43 (1.26) |  |  |  | 23.06 (2.58) |  |  | |  | |  | |
| Time (T1-T0) |  | -0.216 (-0.793, 0.362) | 0.655 |  |  | -0.312 (-0.927, 0.303) | 0.458 | |  | | 0.991 | |
| Time (T2-T0) |  | -0.348 (-0.941, 0.245) | 0.353 |  |  | -0.261 (-0.928, 0.405) | 0.628 | |  | | 0.994 | |
| Time (T2-T1) |  | -0.132 (-0.727, 0.463) | 0.861 |  |  | 0.051 (-0.619 0.721) | 0.983 | |  | | 0.950 | |
| Age: 12 years |  |  |  |  |  |  |  | |  | |  | |
| Baseline | 22.43 (2.92) |  |  |  | 22.77 (2.81) |  |  | |  | |  | |
| Time (T1-T0) |  | -0.166 (-0.758, 0.426) | 0.788 |  |  | -0.112 (-0.668, 0.443) | 0.884 | |  | | 0.998 | |
| Time (T2-T0) |  | -1.017 (-1.669 -0.365) | **<0.001***** |  |  | -0.039 (-0.635, 0.558) | 0.987 | |  | | **0.028*** | |
| Time (T2-T1) |  | -0.851 (-1.499 -0.203) | **0.006**** |  |  | 0.074 (-0.521, 0.668) | 0.955 | |  | | **0.041*** | |
| Age: 13-14 years |  |  |  |  |  |  |  | |  | |  | |
| Baseline | 22.75 (2.05) |  |  |  | 21.61 (4.85) |  |  | |  | |  | |
| Time (T1-T0) |  | 0.250 (-1.174, 1.674) | 0.911 |  |  | 0.267 (-0.906, 1.440) | 0.855 | |  | | 1.000 | |
| Time (T2-T0) |  | -0.691 (-2.323, 0.940) | 0.580 |  |  | -0.506 (-1.827, 0.816) | 0.642 | |  | | 0.996 | |
| Time (T2-T1) |  | -0.941 (-2.573, 0.690) | 0.366 |  |  | -0.773 (-2.056, 0.511) | 0.335 | |  | | 0.997 | |
| Gender: female |  |  |  |  |  |  |  | |  | |  | |
| Baseline | 23.12 (1.99) |  |  |  | 23.10 (2.46) |  |  | |  | |  | |
| Time (T1-T0) |  | -0.290 (-0.759, 0.179) | 0.316 |  |  | -0.158 (-0.636, 0.321) | 0.719 | |  | | 0.955 | |
| Time (T2-T0) |  | -0.653 (-1.152, -0.155) | **0.006**** |  |  | -0.228 (-0.741, 0.285) | 0.551 | |  | | 0.414 | |
| Time (T2-T1) |  | -0.364 (-0.863, 0.135) | 0.202 |  |  | -0.070 (-0.584, 0.444) | 0.945 | |  | | 0.708 | |
| Gender: male |  |  |  |  |  |  |  | |  | |  | |
| Baseline | 23.01 (2.33) |  |  |  | 22.76 (3.13) |  |  | |  | |  | |
| Time (T1-T0) |  | -0.102 (-0.611, 0.407) | 0.885 |  |  | -0.248 (-0.746, 0.250) | 0.473 | |  | | 0.950 | |
| Time (T2-T0) |  | -0.508 (-1.035, 0.019) | 0.062 |  |  | -0.367 (-0.900, 0.166) | 0.239 | |  | | 0.961 | |
| Time (T2-T1) |  | -0.406 (-0.932, 0.120) | 0.167 |  |  | -0.119 (-0.650, 0.412) | 0.858 | |  | | 0.748 | |
| Gender: other or does not want to answer |  |  |  |  |  |  |  | |  | |  | |
| Baseline | 22.60 (1.76) |  |  |  | 21.64 (5.84) |  |  | |  | |  | |
| Time (T1-T0) |  | 0.800 (-0.663, 2.263) | 0.405 |  |  | -2.107 (-3.611, -0.602) | **0.003**** | |  | | **0.004**** | |
| Time (T2-T0) |  | 0.086 (-1.767, 1.938) | 0.994 |  |  | 0.712 (-0.938, 2.363) | 0.569 | |  | | 0.911 | |
| Time (T2-T1) |  | -0.714 (-2.567, 1.138) | 0.637 |  |  | 2.819 (1.178 4.460) | **<0.001***** | |  | | **0.003**** | |
| Friend smokes: no |  |  |  |  |  |  |  | |  | |  | |
| Baseline | 23.23 (1.71) |  |  |  | 22.97 (2.89) |  |  | |  | |  | |
| Time (T1-T0) |  | -0.231 (-0.574, 0.113) | 0.257 |  |  | -0.316 (-0.661, 0.029) | 0.080 | |  | | 0.967 | |
| Time (T2-T0) |  | -0.469 (-0.833, -0.106) | **0.007**** |  |  | -0.216 (-0.586, 0.153) | 0.355 | |  | | 0.582 | |
| Time (T2-T1) |  | -0.239 (-0.602, 0.125) | 0.272 |  |  | 0.100 (-0.269 0.470) | 0.800 | |  | | 0.330 | |
| Friend smokes: yes |  |  |  |  |  |  |  | |  | |  | |
| Baseline | 19.94 (4.77) |  |  |  | 21.31 (4.51) |  |  | |  | |  | |
| Time (T1-T0) |  | 1.183 (-0.265, 2.631) | 0.134 |  |  | -0.051 (-1.448, 1.346) | 0.996 | |  | | 0.387 | |
| Time (T2-T0) |  | -2.116 (-3.640, -0.592) | **0.003**** |  |  | -0.967 (-2.507, 0.574) | 0.305 | |  | | 0.513 | |
| Time (T2-T1) |  | -3.299 (-4.831 -1.767) | **<0.001***** |  |  | -0.915 (-2.423, 0.592) | 0.328 | |  | | **0.028*** | |
| Parent smokes: no |  |  |  |  |  |  |  | |  | |  | |
| Baseline | 23.23 (1.89) |  |  |  | 22.60 (3.47) |  |  | |  | |  | |
| Time (T1-T0) |  | -0.166 (-0.576, 0.244) | 0.609 |  |  | 0.041 (-0.378, 0.459) | 0.972 | |  | | 0.793 | |
| Time (T2-T0) |  | -0.420 (-0.851, 0.010) | 0.057 |  |  | 0.001 (-0.448 0.449) | 1.000 | |  | | 0.300 | |
| Time (T2-T1) |  | -0.254 (-0.684, 0.175) | 0.346 |  |  | -0.040 (-0.489, 0.409) | 0.976 | |  | | 0.803 | |
| Parent smokes: yes |  |  |  |  |  |  |  | |  | |  | |
| Baseline | 22.66 (2.53) |  |  |  | 23.39 (1.83) |  |  | |  | |  | |
| Time (T1-T0) |  | -0.117 (-0.704, 0.471) | 0.887 |  |  | -0.924 (-1.493, -0.356) | **<0.001***** | |  | | 0.061 | |
| Time (T2-T0) |  | -0.859 (-1.490, -0.228) | **0.004**** |  |  | -0.715 (-1.325, -0.105) | **0.017*** | |  | | 0.973 | |
| Time (T2-T1) |  | -0.742 (-1.376, -0.109) | **0.017*** |  |  | 0.210 (-0.397, 0.816) | 0.696 | |  | | **0.033*** | |
| Has tried smoking: no |  |  |  |  |  |  |  | |  | |  | |
| Baseline | 23.20 (1.81) |  |  |  | 23.10 (2.74) |  |  | |  | |  | |
| Time (T1-T0) |  | -0.139 (-0.486, 0.207) | 0.614 |  |  | -0.372 (-0.718, -0.026) | **0.032*** | |  | | 0.603 | |
| Time (T2-T0) |  | -0.465 (-0.831, -0.098) | **0.008**** |  |  | -0.274 (-0.645, 0.097) | 0.193 | |  | | 0.775 | |
| Time (T2-T1) |  | -0.326 (-0.692, 0.041) | 0.093 |  |  | 0.098 (-0.273, 0.468) | 0.810 | |  | | 0.162 | |
| Has tried smoking: yes |  |  |  |  |  |  |  | |  | |  | |
| Baseline | 20.44 (4.47) |  |  |  | 18.57 (4.70) |  |  | |  | |  | |
| Time (T1-T0) |  | -0.342 (-1.749, 1.065) | 0.836 |  |  | 1.071 (-0.446, 2.589) | 0.223 | |  | | 0.293 | |
| Time (T2-T0) |  | -2.002 (-3.510, -0.494) | **0.005**** |  |  | 0.218 (-1.385, 1.821) | 0.946 | |  | | 0.053 | |
| Time (T2-T1) |  | -1.660 (-3.136, -0.184) | **0.023*** |  |  | -0.854 (-2.457, 0.749) | 0.424 | |  | | 0.768 | |
| Lower scores of smoking refusal self-efficacy (≤20) |  |  |  |  |  |  |  | |  | |  | |
| Baseline | 17.66 (2.66) |  |  |  | 15.55 (4.55) |  |  | |  | |  | |
| Time (T1-T0) |  | -0.152 (-0.490, 0.185) | 0.539 |  |  | -0.301 (-0.639, 0.037) | 0.092 | |  | | 0.847 | |
| Time (T2-T0) |  | -0.554 (-0.911, -0.197) | **<0.001***** |  |  | -0.259 (-0.621, 0.104) | 0.215 | |  | | 0.435 | |
| Time (T2-T1) |  | -0.402 (-0.758, -0.045) | **0.023*** |  |  | 0.043 (-0.320, 0.405) | 0.959 | |  | | 0.117 | |
|  |  |  |  |  |  |  |  | |  | |  | |
| T0 = baseline measurement, T1 = 2-week post measurement, T2 = 3-month follow-up measurement  SD = standard deviation, LMM = linear mixed model, CI = confidence interval | | | | | | | | | | | |  |

* p<0.05, ** p<0.01, *** p<0.001

Table 2. Subgroup analysis on sources of smoking refusal self-efficacy among early adolescents

|  | Within group | | | | | | | | |  | | Between group (Group X Time) | | |
| --- | --- | --- | --- | --- | --- | --- | --- | --- | --- | --- | --- | --- | --- | --- |
|  | Control | | |  | | Intervention | | | | |  |  | |  |
|  | Mean (SD) | LMM estimate (95% CI) | P value | |  | | Mean (SD) | LMM estimate (95% CI) | P value | |  | P value | |  |
|  |  |  |  | |  | |  |  |  | |  |  | |  |
| Age: 9-10 years |  |  |  | |  | |  |  |  | |  |  | |  |
| Baseline | 23.24 (2.65) |  |  | |  | | 23.74 (3.09) |  |  | |  |  | |  |
| Time (T1-T0) |  | -0.317 (-1.013, 0.380) | 0.535 | |  | |  | 0.868 (0.123, 1.612) | **0.017*** | |  | **0.019*** | |  |
| Time (T2-T0) |  | -0.682 (-1.402, 0.037) | 0.067 | |  | |  | 0.267 (-0.494, 1.028) | 0.689 | |  | 0.098 | |  |
| Time (T2-T1) |  | -0.366 (-1.085, 0.354) | 0.457 | |  | |  | -0.601 (-1.365 0.164) | 0.156 | |  | 0.936 | |  |
| Age: 11 years |  |  |  | |  | |  |  |  | |  |  | |  |
| Baseline | 23.80 (2.74) |  |  | |  | | 23.35 (3.03) |  |  | |  |  | |  |
| Time (T1-T0) |  | -0.393 (-1.022, 0.237) | 0.309 | |  | |  | -0.200 (-0.865, 0.465) | 0.760 | |  | 0.946 | |  |
| Time (T2-T0) |  | -0.544 (-1.185, 0.098) | 0.115 | |  | |  | -0.917 (-1.645, -0.189) | **0.009**** | |  | 0.746 | |  |
| Time (T2-T1) |  | -0.151 (-0.798, 0.495) | 0.847 | |  | |  | -0.717 (-1.449, 0.015) | 0.056 | |  | 0.437 | |  |
| Age: 12 years |  |  |  | |  | |  |  |  | |  |  | |  |
| Baseline | 22.17 (3.80) |  |  | |  | | 22.88 (2.98) |  |  | |  |  | |  |
| Time (T1-T0) |  | -0.165 (-0.798, 0.468) | 0.813 | |  | |  | 0.165 (-0.441, 0.772) | 0.798 | |  | 0.758 | |  |
| Time (T2-T0) |  | -0.492 (-1.197, 0.214) | 0.231 | |  | |  | -0.069 (-0.712, 0.575) | 0.966 | |  | 0.655 | |  |
| Time (T2-T1) |  | -0.326 (-1.027, 0.374) | 0.518 | |  | |  | -0.234 (-0.875, 0.407) | 0.667 | |  | 0.994 | |  |
| Age: 13-14 years |  |  |  | |  | |  |  |  | |  |  | |  |
| Baseline | 22.25 (2.32) |  |  | |  | | 21.73 (3.10) |  |  | |  |  | |  |
| Time (T1-T0) |  | -0.125 (-1.672, 1.422) | 0.980 | |  | |  | 0.475 (-0.821 1.770) | 0.666 | |  | 0.864 | |  |
| Time (T2-T0) |  | -1.581 (-3.297, 0.136) | 0.079 | |  | |  | 0.102 (-1.360, 1.564) | 0.985 | |  | 0.222 | |  |
| Time (T2-T1) |  | -1.456 (-3.172, 0.261) | 0.115 | |  | |  | -0.372 (-1.768, 1.023) | 0.806 | |  | 0.580 | |  |
| Gender: female |  |  |  | |  | |  |  |  | |  |  | |  |
| Baseline | 23.04 (2.92) |  |  | |  | | 23.04 (2.78) |  |  | |  |  | |  |
| Time (T1-T0) |  | -0.607 (-1.117, -0.097) | **0.015*** | |  | |  | 0.337 (-0.182, 0.855) | 0.280 | |  | **0.007**** | |  |
| Time (T2-T0) |  | -0.809 (-1.350, -0.267) | **0.001**** | |  | |  | -0.329 (-0.883, 0.225) | 0.345 | |  | 0.379 | |  |
| Time (T2-T1) |  | -0.202 (-0.745, 0.342) | 0.659 | |  | |  | -0.666 (-1.220, -0.112) | **0.014*** | |  | 0.408 | |  |
| Gender: male |  |  |  | |  | |  |  |  | |  |  | |  |
| Baseline | 23.07 (3.47) |  |  | |  | | 23.20 (3.27) |  |  | |  |  | |  |
| Time (T1-T0) |  | -0.036 (-0.590, 0.518) | 0.987 | |  | |  | 0.205 (-0.346, 0.756) | 0.658 | |  | 0.851 | |  |
| Time (T2-T0) |  | -0.419 (-0.991, 0.153) | 0.198 | |  | |  | -0.051 (-0.636, 0.534) | 0.977 | |  | 0.644 | |  |
| Time (T2-T1) |  | -0.383 (-0.953, 0.188) | 0.257 | |  | |  | -0.256 (-0.838, 0.327) | 0.558 | |  | 0.977 | |  |
| Gender: other or does not want to answer |  |  |  | |  | |  |  |  | |  |  | |  |
| Baseline | 22.40 (2.61) |  |  | |  | | 23.86 (3.82) |  |  | |  |  | |  |
| Time (T1-T0) |  | 0.867 (-0.731, 2.465) | 0.411 | |  | |  | -0.287 (-1.931, 1.357) | 0.911 | |  | 0.557 | |  |
| Time (T2-T0) |  | -0.446 (-2.473, 1.580) | 0.863 | |  | |  | -0.613 (-2.360, 1.134) | 0.688 | |  | 0.998 | |  |
| Time (T2-T1) |  | -1.313 (-3.339, 0.713) | 0.282 | |  | |  | -0.326 (-2.063, 1.411) | 0.899 | |  | 0.768 | |  |
| Friend smokes: no |  |  |  | |  | |  |  |  | |  |  | |  |
| Baseline | 23.26 (2.88) |  |  | |  | | 23.32 (2.96) |  |  | |  |  | |  |
| Time (T1-T0) |  | -0.330 (-0.705, 0.045) | 0.098 | |  | |  | 0.263 (-0.115, 0.642) | 0.233 | |  | **0.027*** | |  |
| Time (T2-T0) |  | -0.542 (-0.938, -0.147) | **0.004**** | |  | |  | -0.228 (-0.632, 0.175) | 0.380 | |  | 0.473 | |  |
| Time (T2-T1) |  | -0.212 (-0.608, 0.183) | 0.419 | |  | |  | -0.492 (-0.894, -0.089) | **0.012*** | |  | 0.571 | |  |
| Friend smokes: yes |  |  |  | |  | |  |  |  | |  |  | |  |
| Baseline | 18.88 (4.57) |  |  | |  | | 20.53 (3.48) |  |  | |  |  | |  |
| Time (T1-T0) |  | 0.500 (-1.043, 2.044) | 0.727 | |  | |  | 0.000 (-1.497, 1.497) | 1.000 | |  | 0.929 | |  |
| Time (T2-T0) |  | -1.859 (-3.522, -0.196) | **0.024*** | |  | |  | -0.089 (-1.694, 1.516) | 0.991 | |  | 0.202 | |  |
| Time (T2-T1) |  | -2.359 (-4.022, -0.696) | **0.003**** | |  | |  | -0.089 (-1.694, 1.516) | 0.991 | |  | 0.063 | |  |
| Parent smokes: no |  |  |  | |  | |  |  |  | |  |  | |  |
| Baseline | 23.51 (2.83) |  |  | |  | | 23.25 (3.12) |  |  | |  |  | |  |
| Time (T1-T0) |  | -0.461 (-0.906, -0.015) | **0.041*** | |  | |  | 0.343 (-0.117, 0.802) | 0.187 | |  | **0.0098**** | |  |
| Time (T2-T0) |  | -0.647 (-1.113, -0.181) | **0.003**** | |  | |  | -0.098 (-0.587, 0.391) | 0.885 | |  | 0.161 | |  |
| Time (T2-T1) |  | -0.186 (-0.652, 0.280) | 0.616 | |  | |  | -0.441 (-0.930, 0.048) | 0.087 | |  | 0.758 | |  |
| Parent smokes: yes |  |  |  | |  | |  |  |  | |  |  | |  |
| Baseline | 22.02 (3.54) |  |  | |  | | 22.97 (2.96) |  |  | |  |  | |  |
| Time (T1-T0) |  | 0.085 (-0.553, 0.724) | 0.948 | |  | |  | 0.073 (-0.542, 0.689) | 0.958 | |  | 1.000 | |  |
| Time (T2-T0) |  | -0.553 (-1.242, 0.136) | 0.143 | |  | |  | -0.430 (-1.086, 0.227) | 0.274 | |  | 0.986 | |  |
| Time (T2-T1) |  | -0.639 (-1.327, 0.050) | 0.076 | |  | |  | -0.503 (-1.155, 0.149) | 0.167 | |  | 0.981 | |  |
| Has tried smoking: no |  |  |  | |  | |  |  |  | |  |  | |  |
| Baseline | 23.21 (2.92) |  |  | |  | | 23.37 (2.91) |  |  | |  |  | |  |
| Time (T1-T0) |  | -0.327 (-0.703, 0.050) | 0.104 | |  | |  | 0.256 (-0.122, 0.634) | 0.250 | |  | **0.031*** | |  |
| Time (T2-T0) |  | -0.607 (-1.005, -0.210) | **0.001**** | |  | |  | -0.212 (-0.615, 0.191) | 0.434 | |  | 0.274 | |  |
| Time (T2-T1) |  | -0.281 (-0.679, 0.118) | 0.224 | |  | |  | -0.468 (-0.870, -0.066) | **0.018*** | |  | 0.821 | |  |
| Has tried smoking: yes |  |  |  | |  | |  |  |  | |  |  | |  |
| Baseline | 19.88 (4.99) |  |  | |  | | 19.00 (3.04) |  |  | |  |  | |  |
| Time (T1-T0) |  | 0.461 (-1.075, 1.996) | 0.761 | |  | |  | 0.071 (-1.585, 1.728) | 0.994 | |  | 0.969 | |  |
| Time (T2-T0) |  | -0.586 (-2.233, 1.061) | 0.681 | |  | |  | -0.327 (-2.077, 1.423) | 0.899 | |  | 0.992 | |  |
| Time (T2-T1) |  | -1.047 (-2.658, 0.565) | 0.280 | |  | |  | -0.398 (-2.148, 1.352) | 0.854 | |  | 0.891 | |  |
| Lower sources of smoking refusal self-efficacy scores (≤21) |  |  |  | |  | |  |  |  | |  |  | |  |
| Baseline | 18.92 (2.59) |  |  | |  | | 19.25 (1.84) |  |  | |  |  | |  |
| Time (T1-T0) |  | 0.838 (0.026, 1.649) | **0.041*** | |  | |  | 1.453 (0.669, 2.237) | **<0.001***** | |  | 0.488 | |  |
| Time (T2-T0) |  | 0.514 (-0.370, 1.398) | 0.358 | |  | |  | 1.125 (0.272, 1.979) | **0.006**** | |  | 0.565 | |  |
| Time (T2-T1) |  | -0.323 (-1.207, 0.561) | 0.665 | |  | |  | -0.328 (-1.185, 0.529) | 0.640 | |  | 1.000 | |  |
|  |  |  |  | |  | |  |  |  | |  |  | |  |
| T0 = baseline measurement, T1 = 2-week post measurement, T2 = 3-month follow-up measurement  SD = standard deviation, LMM = linear mixed model, CI = confidence interval  * p<0.05, ** p<0.01, *** p<0.001 | | | | | | | | | | | | |  |  |

Table 3. Subgroup analysis on sources of snus refusal self-efficacy among early adolescents

|  | Within group | | | | | | |  | | | Between group (Group X Time) | | |  |
| --- | --- | --- | --- | --- | --- | --- | --- | --- | --- | --- | --- | --- | --- | --- |
|  | Control | | |  | Intervention | | | |  | | |  | | |
|  | Mean (SD) | LMM estimate (95% CI) | P value |  | Mean (SD) | LMM estimate (95% CI) | P value | | |  | | | P value | |
|  |  |  |  |  |  |  |  | | |  | | |  | |
| Age: 9-10 years |  |  |  |  |  |  |  | | |  | | |  | |
| Baseline | 23.03 (2.94) |  |  |  |  |  |  | | |  | | |  | |
| Time (T1-T0) |  | -0.063 (-0.758, 0.631) | 0.975 |  | 23.86 (3.08) | 0.587 (-0.160, 1.335) | 0.156 | | |  | | | 0.352 | |
| Time (T2-T0) |  | 0.024 (-0.694, 0.742) | 0.997 |  |  | -0.144 (-0.922, 0.634) | 0.901 | | |  | | | 0.976 | |
| Time (T2-T1) |  | 0.087 (-0.631, 0.805) | 0.956 |  |  | -0.731 (-1.505, 0.043) | 0.069 | | |  | | | 0.194 | |
| Age: 11 years |  |  |  |  |  |  |  | | |  | | |  | |
| Baseline | 23.74 (3.10) |  |  |  | 23.15 (3.20) |  |  | | |  | | |  | |
| Time (T1-T0) |  | -0.370 (-0.996, 0.256) | 0.348 |  |  | 0.040 (-0.625, 0.706) | 0.989 | | |  | | | 0.645 | |
| Time (T2-T0) |  | -0.570 (-1.215, 0.076) | 0.096 |  |  | -0.308 (-1.027, 0.411) | 0.574 | | |  | | | 0.893 | |
| Time (T2-T1) |  | -0.200 (-0.842, 0.443) | 0.747 |  |  | -0.348 (-1.076, 0.380) | 0.501 | | |  | | | 0.978 | |
| Age: 12 years |  |  |  |  |  |  |  | | |  | | |  | |
| Baseline | 22.38 (3.99) |  |  |  | 22.56 (3.27) |  |  | | |  | | |  | |
| Time (T1-T0) |  | -0.318 (-0.964, 0.327) | 0.479 |  |  | 0.507 (-0.100, 1.115) | 0.123 | | |  | | | 0.085 | |
| Time (T2-T0) |  | -0.802 (-1.512, -0.092) | **0.022*** |  |  | 0.515 (-0.126, 1.156) | 0.143 | | |  | | | **0.004**** | |
| Time (T2-T1) |  | -0.483 (-1.193, 0.227) | 0.247 |  |  | 0.008 (-0.635, 0.651) | 1.000 | | |  | | | 0.542 | |
| Age: 13-14 years |  |  |  |  |  |  |  | | |  | | |  | |
| Baseline | 22.19 (2.64) |  |  |  | 22.09 (2.70) |  |  | | |  | | |  | |
| Time (T1-T0) |  | -0.063 (-1.606, 1.481) | 0.995 |  |  | 0.697 (-0.575, 1.970) | 0.403 | | |  | | | 0.753 | |
| Time (T2-T0) |  | -1.964 (-3.679, -0.249) | **0.020*** |  |  | 0.226 (-1.211 1.664) | 0.928 | | |  | | | 0.064 | |
| Time (T2-T1) |  | -1.902 (-3.616, -0.187) | **0.025*** |  |  | -0.471 (-1.865, 0.923) | 0.707 | | |  | | | 0.339 | |
| Gender: female |  |  |  |  |  |  |  | | |  | | |  | |
| Baseline | 23.07 (2.85) |  |  |  | 22.94 (2.72) |  |  | | |  | | |  | |
| Time (T1-T0) |  | -0.369 (-0.881, 0.143) | 0.208 |  |  | 0.591 (0.068, 1.114) | **0.022*** | | |  | | | **0.006**** | |
| Time (T2-T0) |  | -0.598 (-1.141, -0.055) | **0.027*** |  |  | 0.137 (-0.419, 0.693) | 0.832 | | |  | | | 0.078 | |
| Time (T2-T1) |  | -0.229 (-0.772, 0.314) | 0.583 |  |  | -0.454 (-1.011, 0.103) | 0.135 | | |  | | | 0.873 | |
| Gender: male |  |  |  |  |  |  |  | | |  | | |  | |
| Baseline | 23.09 (3.97) |  |  |  | 23.08 (3.64) |  |  | | |  | | |  | |
| Time (T1-T0) |  | -0.237 (-0.795, 0.321) | 0.579 |  |  | 0.176 (-0.370, 0.723) | 0.730 | | |  | | | 0.516 | |
| Time (T2-T0) |  | -0.563 (-1.140, 0.013) | 0.057 |  |  | 0.073 (-0.512, 0.657) | 0.954 | | |  | | | 0.194 | |
| Time (T2-T1) |  | -0.326 (-0.903, 0.250) | 0.380 |  |  | -0.104 (-0.688, 0.481) | 0.909 | | |  | | | 0.893 | |
| Gender: other or does not want to answer |  |  |  |  |  |  |  | | |  | | |  | |
| Baseline | 21.64 (2.92) |  |  |  | 23.23 (3.61) |  |  | | |  | | |  | |
| Time (T1-T0) |  | 0.875 (-0.764, 2.513) | 0.422 |  |  | 0.546 (-1.199, 2.290) | 0.743 | | |  | | | 0.984 | |
| Time (T2-T0) |  | 0.822 (-1.267, 2.911) | 0.626 |  |  | -0.439 (-2.205, 1.328) | 0.829 | | |  | | | 0.627 | |
| Time (T2-T1) |  | -0.053 (-2.088, 1.983) | 0.998 |  |  | -0.984 (-2.780, 0.812) | 0.403 | | |  | | | 0.806 | |
| Friend smokes: no |  |  |  |  |  |  |  | | |  | | |  | |
| Baseline | 23.22 (3.19) |  |  |  | 23.16 (3.14) |  |  | | |  | | |  | |
| Time (T1-T0) |  | -0.258 (-0.636, 0.120) | 0.245 |  |  | 0.380 (-0.001, 0.762) | 0.051 | | |  | | | **0.016*** | |
| Time (T2-T0) |  | -0.485 (-0.885, -0.085) | **0.013*** |  |  | 0.066 (-0.339, 0.472) | 0.922 | | |  | | | 0.069 | |
| Time (T2-T1) |  | -0.227 (-0.626, 0.172) | 0.377 |  |  | -0.314 (-0.720, 0.092) | 0.165 | | |  | | | 0.978 | |
| Friend smokes: yes |  |  |  |  |  |  |  | | |  | | |  | |
| Baseline | 19.50 (4.65) |  |  |  | 20.82 (3.40) |  |  | | |  | | |  | |
| Time (T1-T0) |  | -0.038 (-1.623, 1.547) | 0.998 |  |  | 0.706 (-0.797, 2.209) | 0.513 | | |  | | | 0.809 | |
| Time (T2-T0) |  | -1.227 (-2.852, 0.398) | 0.179 |  |  | 0.230 (-1.381, 1.842) | 0.940 | | |  | | | 0.353 | |
| Time (T2-T1) |  | -1.190 (-2.853, 0.473) | 0.214 |  |  | -0.476 (-2.087, 1.136) | 0.768 | | |  | | | 0.851 | |
| Parent smokes: no |  |  |  |  |  |  |  | | |  | | |  | |
| Baseline | 23.46 (3.22) |  |  |  | 23.05 (3.31) |  |  | | |  | | |  | |
| Time (T1-T0) |  | -0.554 (-1.001, -0.108) | **0.010*** |  |  | 0.482 (0.027, 0.938) | **0.035*** | | |  | | | **<0.001***** | |
| Time (T2-T0) |  | -0.667 (-1.135, -0.199) | **0.003**** |  |  | -0.010 (-0.496, 0.476) | 0.999 | | |  | | | 0.066 | |
| Time (T2-T1) |  | -0.113 (-0.581, 0.355) | 0.839 |  |  | -0.492 (-0.978, -0.006) | **0.046*** | | |  | | | 0.462 | |
| Parent smokes: yes |  |  |  |  |  |  |  | | |  | | |  | |
| Baseline | 22.08 (3.56) |  |  |  | 22.95 (2.98) |  |  | | |  | | |  | |
| Time (T1-T0) |  | 0.376 (-0.266, 1.018) | 0.355 |  |  | 0.243 (-0.383, 0.868) | 0.634 | | |  | | | 0.980 | |
| Time (T2-T0) |  | -0.244 (-0.931, 0.443) | 0.683 |  |  | 0.242 (-0.420, 0.904) | 0.667 | | |  | | | 0.548 | |
| Time (T2-T1) |  | -0.620 (-1.305, 0.065) | 0.086 |  |  | -0.001 (-0.665, 0.664) | 1.000 | | |  | | | 0.338 | |
| Has tried smoking: no |  |  |  |  |  |  |  | | |  | | |  | |
| Baseline | 23.07 (3.33) |  |  |  | 23.25 (3.00) |  |  | | |  | | |  | |
| Time (T1-T0) |  | -0.245 (-0.622, 0.132) | 0.279 |  |  | 0.376 (-0.003, 0.755) | 0.052 | | |  | | | **0.019*** | |
| Time (T2-T0) |  | -0.458 (-0.858, -0.058) | **0.020*** |  |  | 0.082 (-0.322, 0.485) | 0.883 | | |  | | | 0.076 | |
| Time (T2-T1) |  | -0.213 (-0.612, 0.186) | 0.421 |  |  | -0.295 (-0.698, 0.109) | 0.201 | | |  | | | 0.982 | |
| Has tried smoking: yes |  |  |  |  |  |  |  | | |  | | |  | |
| Baseline | 22.06 (4.25) |  |  |  | 18.57 (3.65) |  |  | | |  | | |  | |
| Time (T1-T0) |  | -0.245 (-1.899, 1.410) | 0.936 |  |  | 0.857 (-0.798, 2.512) | 0.444 | | |  | | | 0.610 | |
| Time (T2-T0) |  | -1.598 (-3.211, 0.014) | 0.053 |  |  | 0.053 (-1.696, 1.802) | 0.997 | | |  | | | 0.280 | |
| Time (T2-T1) |  | -1.354 (-3.016, 0.309) | 0.136 |  |  | -0.804 (-2.553, 0.945) | 0.528 | | |  | | | 0.933 | |
| Lower sources of snus refusal self-efficacy scores (≤21) |  |  |  |  |  |  |  | | |  | | |  | |
| Baseline | 18.79 (2.64) |  |  |  | 19.31 (1.86) |  |  | | |  | | |  | |
| Time (T1-T0) |  | -0.245 (-0.613, 0.122) | 0.261 |  |  | 0.400 (0.031, 0.769) | **0.030*** | | |  | | | **0.011*** | |
| Time (T2-T0) |  | -0.529 (-0.917, -0.141) | **0.004**** |  |  | 0.075 (-0.318, 0.468) | 0.894 | | |  | | | **0.031*** | |
| Time (T2-T1) |  | -0.284 (-0.672, 0.104) | 0.199 |  |  | -0.325 (-0.718, 0.069) | 0.129 | | |  | | | 0.997 | |
|  |  |  |  |  |  |  |  | | |  | | |  | |
| T0 = baseline measurement, T1 = 2-week post measurement, T2 = 3-month follow-up measurement  SD = standard deviation, LMM = linear mixed model, CI = confidence interval  * p<0.05, ** p<0.01, *** p<0.001 | | | | | | | | | | | | | | |
